# Health surveillance representative of koala (*Phascolarctos cinereus*) distribution in Victoria, Australia

**DOI:** 10.1111/avj.13208

**Published:** 2022-10-19

**Authors:** M Cooley, P Whiteley, G Thornton, M Stevenson

**Affiliations:** ^1^ School of Veterinary Medicine Royal Veterinary College Hatfield Hertfordshire AL9 7TA UK; ^2^ Melbourne Veterinary School, Faculty of Veterinary and Agricultural Sciences The University of Melbourne Werribee Victoria 3030 Australia; ^3^ Melbourne Veterinary School The University of Melbourne Melbourne Victoria 3010 Australia

**Keywords:** citizen science, Koala, observation, standardised surveillance ratio, surveillance, syndromic surveillance

## Abstract

Health surveillance of wildlife populations is essential for conservation and reduction of the impacts of disease. Population declines and areas of overabundance of koalas (*Phascolarctos cinereus*) can disrupt the overall survival of the species as well as its habitat. This retrospective study was conducted to describe population distributions, identify areas which need increased surveillance and improve koala health surveillance methodology by Wildlife Health Victoria: Surveillance (WHV:S) at the Veterinary School of The University of Melbourne. Twelve years of Victorian koala observation data from the Atlas of Living Australia combined with surveillance data from WHV:S were used to create choropleth maps, using Quantum Geographic Information Systems of populations and surveillance events, visually representing hot spots. This data was further used to calculate health surveillance efforts between 2008 to the beginning of 2020. Analysis ranked postcodes throughout Victoria from low surveillance efforts to high, using standardised surveillance ratio's 95% confidence interval upper limits which were mapped using a colour gradient. This identified postcodes which need increased surveillance effort, corresponding to areas with high koala observations and low surveillance submissions. This analysis can guide surveillance for postcodes with koalas that were under‐represented and inform improved methodology of future surveillance by WHV:S. The specific advice for improvements to WHV:S includes utilisation of citizen science and syndromic surveillance, website improvement, increasing community awareness and more. The limitations of this study were discussed.

**Abbreviations**
ALA: Atlas of Living Australia
CWHC: Canadian Wildlife Health Cooperative
DELWP: (Victorian) Department of Environment, Land, Water and Planning
KoRV: Koala retrovirus
OIE: Organization International des Epizooties, World Organisation for Animal Health
SSR: standardised surveillance ratio
WHA: Wildlife Health Australia
WHV:S: Wildlife Health Victoria: Surveillance

The koala (*Phascolarctos cinereus*), an arboreal marsupial that feeds on eucalyptus species, is a worldwide symbol of Australia's wildlife.^1^ In a study conducted in 2014 by Conrad and colleagues for the Australian Koala Foundation, it was estimated that koalas generate in the order of AUD 3.2 billion annually and are associated with approximately 30,000 jobs. Most of these jobs are throughout the Australian tourism industry.^2^ During the time of hunting of koalas, over 3 million pelts went to market in the early 1900's.^3^ In 2020, The (Victorian) Department of Environment, Land, Water and Planning (DELWP) estimated there were 413,000 koalas in Victoria. Despite the massive contributions of the koala to Australia's cultural and economic prosperity, its populations have been threatened since the late 1800's. These threats include, but are not limited to hunting (up to late 1900's),^2^, ^4^ loss of habitat,^2^, ^5^, ^6^ road‐traffic‐related incidents, animal attacks,^6^ bushfires,^7^ low genetic diversity due to restocking from small island populations in the early 1900s,^8^, ^9^ climate change (including drought) and infectious diseases.^10^, ^11^, ^12^

The main diseases that impact koalas include chlamydiosis, sarcoptic mange and various opportunistic bacterial (*Bordetella bronchiseptica*, *Corynebacterium* spp., etc.), fungal (*Aspergillus* sp., dermatomycosis) and parasitic diseases (toxoplasmosis, *Sarcoptes scabiei*, etc.).^6^, ^13^ *Chlamydia* spp. infects ocular and urogenital tissues, which can cause infertility, progressive blindness and general debility. There is increasing evidence that *Chlamydia percorum* has come from introduced sheep, cattle, or pigs as a result of colonisation, which has been detected in Victorian koala populations.^14^, ^15^ Koalas may also be infected with koala retrovirus (KoRV). Koala retrovirus, a *gammaretrovirus*, has five subtypes.^6^, ^14^ In Queensland and Northern New South Wales, KoRV is vertically transmitted^16^ and thought to be associated with an increased incidence of lymphocytic neoplasia, and immunosuppression, potentially making them more susceptible to developing chlamydial disease.^16^ The complicated genetic sequence and dynamic nature of KoRV have made the relationship between disease and viral load unclear and is currently under investigation.^17^

Persistently and concurrently, koalas can be impacted by a variety of parasitic, bacterial, viral and fungal diseases. The majority of reports on disease agents such as *Toxoplasma*, *Cryptococcus*, *Aspergillus* and more come from studies on captive koalas, which is not within the scope of this study.^12^ Identifying the impact of infectious diseases on wildlife populations is difficult as koala abundance and population trends are hard to monitor and vary geographically. It has been suggested that infectious disease may play a role in population declines.^6^, ^9^, ^11^, ^14^, ^18^ Despite these claims, there has been weak association between population declines and infectious disease, leaving poor evidence to suggest population impacts of diseases such as *Chlamydia*.^4^, ^10^ According to McCallum,^4^ increased stress can make koalas more susceptible to succumbing to diseases they come in contact with, and because they are under stress‐less tolerant or able to fight off infection once they encounter the disease, which then leads to a decline in population numbers.^4^ These considerations are important when analysing wildlife populations at risk.^4^, ^10^ It is also important to take into account causes of premature death in koalas when analysing population data. Premature deaths in koalas can be attributed to anything other than old age or dying a natural death. These causes include traumatic injuries by motor vehicle collisions and animal attacks, stochastic events such as bushfires, floods, droughts and loss of habitat due to overabundance, climate change and intentional deforestation for buildings and development.^6^, ^19^, ^20^

Animal‐health surveillance helps to inform decisions which help to reduce the impact of animal diseases.^21^ While koala populations have experienced threats throughout history, it is wise to improve ongoing surveillance practices to monitor the health status of populations and to better understand factors influencing turnover and the epidemiology of disease threats. Disease can affect mortality and morbidity, reproduction and fitness, and new diseases can occur through changes in existing diseases, spill‐over of new infections from other animal species, reduced koala genetic diversity impacting immune function, disease resistance, reproduction and ability to adapt to change. Without surveillance, these disease threats can go undetected, and so surveillance is part of mitigation strategies informing and following disease risk analyses.

The World Organisation for Animal Health (OIE) defines surveillance as the ‘systemic on‐going collection, collation and analysis of information related to animal health and the timely dissemination of information so that action can be taken’.^22^ Surveillance is imperative to determine the distribution of current diseases and detect the early emergence of novel diseases in wildlife.^23^ Within Australia, the OIE receives information from Wildlife Health Australia (WHA).^13^ Information from WHA is derived from a variety of sources, including Wildlife Health Victoria: Surveillance (WHV:S), a programme developed in 2008. WHV:S has a bottom‐up focus based on collaboration and cooperation with community groups (Landcare, wildlife carers, bird observers, field naturalists etc.), staff of the Victorian Department of Environment, Land Water and Planning, Parks Victoria, Agriculture Victoria, Zoos Victoria, Environmental Protection Agency Victoria, other universities working together to create a network of wildlife health information. The model used by WHV:S is derived from the Canadian Wildlife Health Cooperative (CWHC) which links Canada's Veterinary Colleges together and was established in 1990.^24^ Since 2008, WHV:S has contributed to wildlife disease surveillance in Victoria through partnership with WHA and contributed to research at the Melbourne Veterinary School resulting in 10 peer reviewed publications. The first report on sarcoptic mange in free‐ranging koalas documented an emerging disease impacting wildlife.^13^ In 2020, surveillance investigations led to a report on oxalate nephrosis in koalas in Victoria.^25^ This surveillance improves baseline knowledge and is important for a number of reasons. Early identification of emerging diseases allows cost‐efficient and rapid methods, if available, for mitigating population impacts to be applied. In turn, this improves biosecurity, minimises the likelihood of spill over of disease to other endemic and feral wildlife, domestic animals and promotes a higher standard of One Health throughout Australia.

Analysis of the current and retrospective work by WHV:S can provide insight into successful areas as well as what aspects of surveillance and mitigation strategies need to be improved. An internal review by Thornton^23^ and Whiteley was undertaken reviewing the first 9 years of WHV:S's existence (2008–2016), analysing its performance and comparing it to its predecessors such as the CWHC and OIE. The 2017 study shed light on gaps in knowledge such as discrepancies in locations of submissions due to geographical bias. Limited resources such as funding streams and staffing can impact data collection efforts. A paper published by the CWHC details twenty‐first century challenges to wildlife health.^24^ The paper by Stephen (2015) outlines the increased effectiveness of social media and citizen science in wildlife health surveillance, which could be a potential path of exploration by WHV:S to maximise resources. Citizen science is the practice of public collaboration of data monitoring and collection, with the purpose of contributing to scientific knowledge.^26^, ^27^ The Australian Museum is currently undergoing multiple citizen science programmes to monitor various species as well as a photographic monitoring programme to document climate change. The Victorian DELWP called on citizen scientists to participate in a ‘koala count’ on a single day in 2015 using a smartphone application available for iOS and Android. Citizen science counts, collected by the application, were then uploaded to ALA and Victorian Biodiversity Atlas. This is a prime example of citizen science programmes that should be engaged for improvement of koala population data.

The aims of this study were to: (1) describe the geographic distribution of koala populations and surveillance efforts across Victoria for 2008–2020 using data from Atlas of Living Australia (ALA) and WHV:S for 2008, 2010–2020, (2) Analyse data and calculate standardised surveillance ratios (SSRs) to identify postcodes which need increased surveillance effort and (3) Provide a representative sampling approach for future koala health surveillance effort in Victoria.

## Materials and methods

Two primary data sets were analysed. The first provided details of dead koalas investigated by WHV:S for the period January 2008 to February 2020 (n = 257 records). Details for each necropsied koala included specimen pathology number, location of acquisition (as a postcode), body weight, sex and cause of death as recorded at the time of necropsy. The second data set was from ALA, which included details of observations of koalas reported by the general public in Victoria for 2008 and 2010–2020 (n = 1068 sighting events); no data were available for 2009. The ALA data included the date and location details for each koala observed. Koala sighting location details (in decimal degree format) were converted to Australian four‐digit postcode area identifiers using tools provided by latlong.net.

Choropleth maps were developed to show counts of WHV:S koala submissions by postcode area for the 11‐year period, 2008 and 2010–2020. Instructional videos were used to create choropleth maps.^28^ ALA data were used to create a choropleth map showing the total number of koala observations per postcode area for the same time period. Using this approach, the WHV:S data provided an estimate of the outcome of interest for this study (the geographic distribution of koala surveillance cases by postcode area) and the ALA data provided a proxy measure of the geographic distribution of the koala population at risk for the same time period. An implicit assumption in use of this approach is that the ALA data provide an accurate (but not precise) estimate of the geographic distribution of the koala population at risk in Victoria.

A spreadsheet was created listing, for each postcode area, the total number of WHV:S surveillance events for 2008, 2010–2020 and the total number of ALA koalas observed in each postcode area for 2008, 2010–2020. Once this was done, the total number of koalas observed in each of the postcode areas and the total number of WHV:S koala surveillance cases in each of the postcode areas were summed to return $T_{\mathrm{pop}}$ and $T_{\mathrm{obs}}$, respectively. $T_{\mathrm{obs}}$ was then divided by $T_{\mathrm{pop}}$ as an estimate of what is termed the state‐wide surveillance fraction for the 12‐year study period. The state‐wide surveillance fraction was multiplied by the number of koalas observed in each of the postcode areas to provide the expected number of WHV:S surveillance events for each postcode area. Finally, the observed number of WHV:S surveillance events for each postcode area was divided by the expected number to return a postcode area level SSR. A 95% confidence interval (CI) around each postcode area SSR estimate was calculated using Open Epi (URL: https://www.openepi.com/SMR/SMR.htm).

Calculation of SSR:

$$T_{\mathrm{obs}}=\text{total number of surveillance events}\;\left(\mathrm{WHV}:S\right)$$

$$T_{\mathrm{pop}}=\text{total number of observations}\;\left(\mathrm{ALA}\right)$$

$$\frac{T_{\mathrm{obs}}}{T_{\mathrm{pop}}}=\text{State wide surveillance fraction}$$

State wide surveillance fraction*koala observationsbypostcode=Expected number of koalas in each post code

$$\frac{\text{Total}\;\mathrm{WHV}:\text{surveillance events}}{\text{Expected number of koalas in each postcode}}=\mathrm{SSR}\;\mathrm{by}\;\text{postcode}\;\left(+/-\mathrm{CI}\right)$$

## Results

### Distribution of koala populations

The purpose of this pilot study was to (1) describe the geographic distribution of koala populations across Victoria. WHV:S necropsy submissions combined with ALA observation data from 2008, 2010–2020 were representative of these populations. The ALA data were used to determine presence of koalas, and WHV:S data were used to determine surveillance sampling. (2) Analyse data and calculate SSRs to identify postcodes which need increased surveillance effort. (3) Provide a representative sampling approach for future koala health surveillance effort in Victoria.

Table 1 summarises the geographical variation in specimen submissions to WHV:S and ALA koala observations. The highest four postcodes were chosen from each set of data. The postcodes with the highest ALA observations were 3233 (*Cape Otway*, *Marengo*, *Apollo Bay*, *Petticoat Creek*, *Skenes Creek North*), 3221 (*Barrabool*, *Gnarwarre*, *Ceres*), 3304 (*Heywood*, *Bessiebelle*, *Dartmore*, *etc*) and 3303 (*Condah, etc*). The postcodes with highest WHV:S submissions were 3233 (*Cape Otway*, *Marengo*, *Apollo Bay*, *Petticoat Creek*, *Skenes Creek North*), 3880 (*Raymond Island*, *etc*), 3641 (*Mywee*, *Ulupna*, *Strathmerton*, *etc*) and 3921 (*French Island*).

**Table 1:** Geographical koala submissions to Wildlife Health Victoria: Surveillance (WHV:S) and Atlas of Living Australia (ALA)

Post code areas	WHV:S submissions	ALA observations
3233 (Cape Otway)	42	96
3221 (Ceres)	—	85
3304 (Heywood, Dartmoor)	—	64
3303 (Condah)	—	55
3880 (Raymond Island)	31	—
3641 (Mywee)	14	—
3921 (French Island)	13	—

Highest four postcodes for WHV:S submissions and ALA observations.

Table 2 summarises the annual variation in specimen submissions to WHV:S and ALA koala observations. The year with the greatest number of submissions to WHV:S was 2013. The year with the highest number of ALA observations was 2015. Since 2008, WHV:S has received 257 submissions varying widely between each year. The least number of submissions occurred in 2008, 2017 and 2020. 2020 submissions only included up until February, as this was when data were analysed. ALA also has a stratified submission number annually. The years with highest observations were 2013–2015, while 2009 had the lowest.

**Table 2:** Annual variation in koala submissions to Wildlife Health Victoria: Surveillance (WHV:S) and Atlas of Living Australia (ALA) observations

Year	WHV:S koala submissions	ALA koala observations
2008	4	53
2009	7	0
2010	31	27
2011	29	48
2012	31	91
2013	71	134
2014	18	182
2015	17	264
2016	14	37
2017	4	42
2018	15	60
2019	12	96
2020	4	33
Total	257	1068

**Figure 1:**
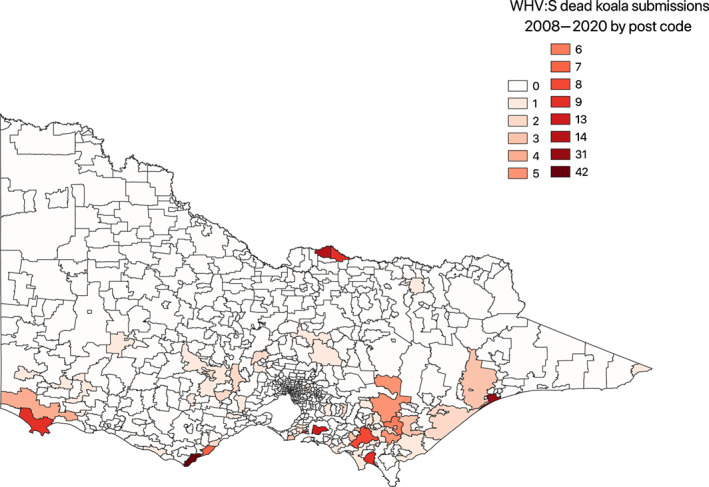
Distribution of koalas submitted to Wildlife Health Victoria: Surveillance (WHV:S) for the period of 2008–2020 by postcode. Digital map sourced from the University of Melbourne spatial catalogue (https://unimelb.libguides.com/GIS/base). Chloropleth map generated using Quantum Geographic Information System.

Figure 1 is a visual representation of all postcodes in Victoria, highlighting WHV:S koala submission data from 2008–2020. The postcodes with highest numbers of observations in dark red, and the lowest areas in white representing zero submissions.

**Figure 2:**
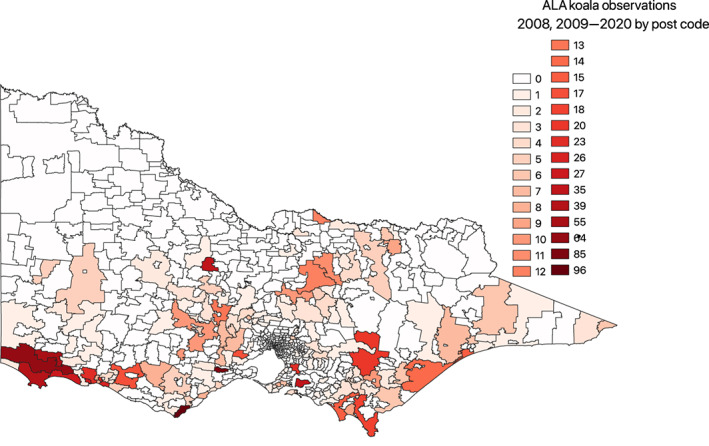
Distribution of koala observations submitted to Atlas of Living Australia (ALA) for the period 2008–2020 by postcode. The data used to produce this map was sourced from the ALA data (https://www.ala.org.au/). Digital map sourced from the University of Melbourne spatial catalogue (https://unimelb.libguides.com/GIS/base). Chloropleth map generated using Quantum Geographic Information System.

Figure 2 is a visual representation of all postcodes in Victoria, highlighting ALA observation data from 2008–2020. The postcodes with highest numbers of observations in dark red, and the lowest areas in white representing zero submissions.

**Figure 3:**
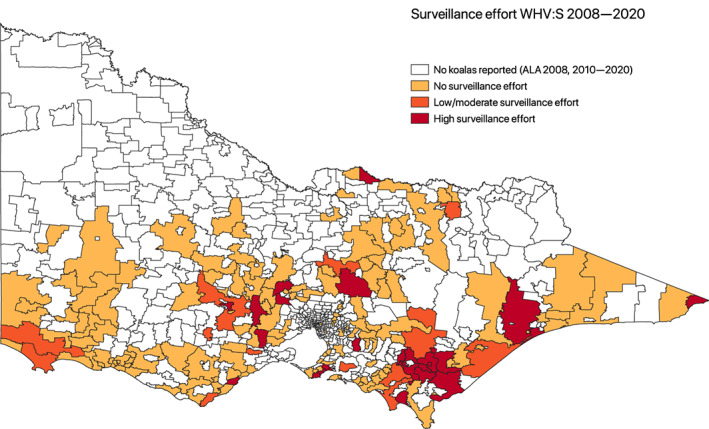
Koala surveillance effort across Victoria by postcode. Standard surveillance ratio's by postcode for 2008–2020 (95% confidence intervals upper limits). Digital map sourced from the University of Melbourne spatial catalogue (https://unimelb.libguides.com/GIS/base). Chloropleth map generated using Quantum Geographic Information System. WHV:S, Wildlife Health Victoria: Surveillance.

Figure 3 is a visual representation of areas within Victoria with high–low surveillance effort. Areas with no resident koalas are reported in white, with areas of no current surveillance in light orange, areas with low–moderate surveillance effort in orange, and areas of high relative surveillance effort in red. Areas in light orange are identified as areas which need increased surveillance effort in the future.

## Discussion

### Distribution of koala populations

Evaluation of distributions of koala populations and koala surveillance events was mapped by postcode for Victoria. The highest four postcodes from each set of data were summarised in Table 1. The north‐west region of Victoria corresponds to areas where koalas have not been reported, which is also confirmed by other large‐scale studies done by DELWP.^5^ These areas correspond with an absence of suitable koala habitat.^19^ Observation of koala data may also be missing in areas with low observer density. Furthermore, with modern technology such as listening for koala calls, scat surveys and increased funding, the remnant areas can be more thoroughly examined for the presence of koalas. Understanding habitat dynamics was important in evaluation of koala populations.^19^ In locations such as Cape Otway, the carrying capacity was borderline exceeded, resulting in degradation of eucalypt forests, having a negative cascade effect on koala morbidity and mortality.^19^ Overabundant koala populations were part of a management programme at Mt Eccles (postcode 3286, Bessiebelle), Cape Otway, Raymond Island and French Island.^1^, ^19^ These four areas are noted in WHV:S koala surveillance cases as euthanased koalas. Various ongoing ecological studies have contributed concentrated surveillance efforts and as a result were ranked as areas of higher surveillance effort.

Influences on koala populations appear to be driven by a multitude of dynamic factors changing spatially and temporally. Studies indicate that the role of disease is unclear and needs further research.^4^ Events such as bushfires and drought have negative impacts on koalas, whereas translocations can help to reduce overabundance and positively impact populations.^4^, ^20^ Droughts and heatwaves have been linked to population declines and ill‐health in koalas and can be used as an indicator for periods of koalas deaths and decreased birth rates.^20^ Climate change causes increased carbon dioxide in the environment, which allows for less available resources for *Eucalyptus* to grow and prosper, further threatening their natural habitat.^20^ A recently published ‘Koala Recovery Plan’ has been introduced for the regions of Queensland, New South Wales and the Australian Capital Territory which identified the timely need for intervention to renew koala populations.^18^ While this study does not address Victoria, its underlying concepts are still relevant and applicable. The study identifies the main direct threats as climate change, land use changes, dogs and vehicles and disease.^18^

The data collected in this study may not reflect the distribution or abundance of koalas in Victoria and is dependent on observation. In a citizen science study done in 2019 in Queensland by Dissanayake and colleagues, it was concluded that sightings were influenced by breeding season, as well as increased in residential, agricultural and parkland areas, showing a decrease in sightings further away from primary roads.^27^

Using a compilation of data and statistical efforts, a list of postcodes with low surveillance effort was successfully created. The calculated SSR is derived from the epidemiological calculation of standard mortality ratios, but for this study, it was representative of surveillance, instead of mortality. Providing an indirect method of standardisation allowed the surveillance effort to be quantified with respect to the general population. This was important for our study because it represented a ranking of surveillance efforts, which were mapped on a colour gradient using 95% CIs (Figure 3). Postcodes with no surveillance effort were white, low–moderate surveillance effort in light orange (SSR 0–1) and those in orange (SSR values 1–2.9) were identified as postcodes with high surveillance effort. Postcodes with low WHV:S submissions and high ALA observations are representative of areas with low‐surveillance effort despite the presence of koala populations where increased community awareness of WHV:S may improve koala health surveillance. Table 1 displays provide a guideline for top postcodes worth directing efforts towards in the immediate future due to the highest discrepancy between populations and surveillance events. These postcodes include 3221 (Ceres West of Geelong), 3304 (Heywood, Dartmore) and 3303 (Condah).

Postcodes with high WHV:S submissions and low ALA observations (e.g., 3641) could be a reflection of low human populations or possibly high numbers of active wildlife carers (two very active wildlife carers) or diligent veterinary practitioners (local veterinarian and diagnosis by Healesville Sanctuary veterinarian, Zoos Victoria).

### Review of WHV surveillance programme

The final aim of this study was to evaluate the methodology of WHV surveillance of koala populations in Victoria. This surveillance has been very basic to date. WHV:S is relatively new programme with limited resources and is working to continue to improve methodology and expand their resource network. However, WHV:S has investigated koala health^29^ and published on sarcoptic mange^13^, ^30^, ^31^ and oxalate nephrosis,^25^ and contributed to research into Chlamydia,^14^ Koala Retrovirus^32^ and Herpesvirus^33^, ^34^, ^35^ in koalas.

The years with the most submissions were 2013 with 71 submissions, followed by 2012 and 2010 with 31 and 2011 with 29 (Table 2). This was not the only factor used to evaluate success and failures of WHV:S but also provides insight. It is unclear why these years have increased submissions although each year, the surveillance effort varied for a variety of reasons linked to limited resources and focusing on key wildlife health issues, which is reflected in the submission data. The number of ALA observations peaked in 2015 with 264 koala sightings. This was a year with particularly concentrated effort, as this was the year the ALA ‘Victorian Great Koala Count’ took place. This demonstrates the value of this programme to improve knowledge of the distribution and abundance of koalas in Victoria and potentially population trends. The results should be interpreted with caution, as they are only derived from limited data provided and, in an effort, to establish a baseline. These data sets are not entirely comprehensive but bring researchers closer to understanding current koala populations. The data can reflect the results of various projects with concentrated efforts, such as the management efforts in Cape Otway, Mt Eccles, French and Raymond Islands.

Collection of wildlife sighting data requires funding and resources. Infrequent collection of data is only representative of isolated geographical areas and time periods.^27^ The work of the community is invaluable to the surveillance of koalas, as well as other species throughout the world. Contributions from the community through citizen science can improve population counts. Increasing training and awareness of the importance of wildlife surveillance throughout the community will be useful as well.^21^ The validation of citizen science is discussed in various studies, and it has been shown, it can be used to refine distribution and population estimates.^27^ In addition to citizen science, syndromic surveillance could allow for more directed surveillance and allow a better dispersion of already limited resources. Syndromic surveillance is defined by the World Health Organization as ‘the continuous, systematic collection, analysis and interpretation of health‐related data needed for the planning, implementation, and evaluation of public health practice’. It can be used as an indicator and assists in early detection of emerging disease which may impact wildlife populations. A survey or webpage with lists of potential syndromes or clinical signs could be distributed to the general‐public as well as veterinary practices and wildlife carers. Furthermore, stakeholders such as carers and vets can be further trained and supported in the gathering of data. WHV:S currently has ethical approval to obtain samples from wildlife which are under anaesthetic for other procedures. Frequently, wildlife is brought into vet practices to receive care but are not documented or sampled for disease. This can be a valuable resource to target for increased general/scanning surveillance samples. This information could allow for more targeted surveillance projects to be conducted. It would be advantageous to focus on veterinary practices with high numbers of observations but low dead koala submissions. Some funding support is available to Veterinary Practitioners from the National Significant Disease Investigation programme.^36^ It would benefit WHV:S to work with these practices to establish a protocol of sampling and continue to work with each practice by providing materials to sample wildlife. In addition to aforementioned suggestions, a greater focus should be placed on gathering and utilising data from rehabilitator groups to improve record keeping for disease and causes of premature death, as discussed in a recent paper by Lunney et al.^37^

Brief specific plans for the immediate future should also include adopting a standardised necropsy procedure, which will assist in the diagnosis of mortality for koalas submitted to WHV:S in future. This can help to detect large clusters of specific diseases. Improvement of the WHV:S website could also lead to improved submission data and user friendliness to spark interest from potential financier's and partners. Increased funding would enable more overnight shipment of dead koalas for diagnosis at the Melbourne Veterinary School and cover costs such as making tissue microscope slides for histopathological diagnosis.

In addition to citizen science, the implementation of syndromic surveillance should be considered to improve koala surveillance by WHV:S. The US Centre for Disease Control and Prevention defines the ‘fundamental objective of syndromic surveillance is to identify illness clusters early, before diagnoses are confirmed and reported to public health agencies, and to mobilise a rapid response, thereby reducing morbidity and mortality’.^38^ Syndromic surveillance relies on the observation and reporting of clinical signs, possibly animal numbers and environmental measures. The advantage of syndromic surveillance reporting is it is fast and inexpensive. It aims to detect outbreaks, which can be used for follow‐up general surveillance by trained professionals who can provide diagnosis and disease agent identification. These various practices have been touched on by WHV:S, submitting surveys to various stakeholders, but further time and development of methodology and infrastructure should be pursued. With funding, WHV:S can contribute to a disease risk analysis to identify priority diseases, mitigation strategies and gaps for koalas and an ongoing, improved surveillance system in Victoria.

## Conclusions

The purpose of this study was to (1) collate data and create a visual representation of geographic distributions of koala populations in Victoria for 2008–2020, (2) use this collated data to develop a list of postcodes in need of increased surveillance effort and (3) to evaluate WHV:S as a surveillance programme and highlight areas for improvement in methodology.

Using choropleth maps allowed visual representation for koala surveillance death and koala observation hot spots. Areas of high populations were identified in both data sets. A baseline for postcodes of koalas at risk was developed, as well as areas which Victoria's koala populations are distributed. The comprehensiveness of the choropleth maps was gauged by investigating WHV:S as a surveillance programme. A map of SSR values visually ranks surveillance efforts from low to high. Future surveillance should focus on areas of low surveillance effort. While not the focus of this study, understanding potential influences on koala populations, such as diseases, low genetic diversity, koala habitat quality and corridors and more, these maps can be better understood. The study relied greatly on retrospectively collected data from sources with limited funding and resources. The limitations of the data are extensive, but one of importance comes with general surveillance. The distribution of observation and mortality data collection contains bias. In areas with more humans to send in the data, there are more submissions and observations, and less in the sparsely populated areas. Other source of bias, for example, could be koala management efforts or active veterinarians and wildlife carers submitting data to WHV:S which could have created unprecedented numbers in specific postcodes. The implications of periodic data assemblage and reflection, such as this study, aim to progress koala population profiles. Additional investigations using areas with high dead koala submissions can be targeted for more in‐depth surveillance and active surveillance programmes. Syndromic surveillance reporting can be used to trigger general surveillance efforts. These studies can further investigate causes of morbidity and mortality. Also, by re‐evaluating the methodology of WHV:S, refinements can be made. Considerations for methods of improvement include utilising all relevant stakeholders to gather quality information, implementation of syndromic surveillance and citizen science data collection efforts, improvement of community awareness, website improvement, as well as pursuit of increased funding options.

## Conflict of interest and sources of funding

The authors declare no conflicts of interest or sources of funding for the work presented here.

## Supporting information


**Figure S1.** Koala observations ALA by postcode.

**Figure S2.** Koala observations WHV:S submissions by postcode.

**Figure S3.** SSR for each postcode.
