# Supplementary material for: Health surveillance representative of koala (Phascolarctos cinereus) distribution in Victoria, Australia
Source: Aust Vet J. 2022 Oct 19;100(12):605–12. doi: 10.1111/avj.13208 (PMC10092863; doi:10.1111/avj.13208)
Supplement: Supplementary file 1 — Figure S1. Koala observations ALA by postcode. [file AVJ-100-605-s002.docx]

Figure 1 Supplementary information: Koala Observations ALA by postcode

| **number of observations** | **post code** |
| --- | --- |
| **1** | 2714 |
| **1** | 3024 |
| **6** | 3097 |
| **1** | 3101 |
| **2** | 3113 |
| **1** | 3114 |
| **2** | 3132 |
| **3** | 3139 |
| **1** | 3156 |
| **1** | 3158 |
| **1** | 3166 |
| **1** | 3183 |
| **1** | 3196 |
| **1** | 3199 |
| **17** | 3211 |
| **1** | 3212 |
| **1** | 3213 |
| **85** | 3221 |
| **5** | 3230 |
| **3** | 3231 |
| **8** | 3232 |
| **96** | 3233 |
| **1** | 3235 |
| **1** | 3236 |
| **2** | 3238 |
| **1** | 3239 |
| **1** | 3241 |
| **5** | 3249 |
| **8** | 3260 |
| **17** | 3265 |
| **1** | 3275 |
| **5** | 3277 |
| **1** | 3278 |
| **3** | 3279 |
| **26** | 3280 |
| **4** | 3281 |
| **8** | 3282 |
| **23** | 3283 |
| **2** | 3284 |
| **27** | 3285 |
| **2** | 3286 |
| **1** | 3289 |
| **2** | 3292 |
| **1** | 3294 |
| **1** | 3300 |
| **55** | 3303 |
| **64** | 3304 |
| **39** | 3305 |
| **1** | 3312 |
| **1** | 3314 |
| **2** | 3321 |
| **7** | 3331 |
| **7** | 3340 |
| **5** | 3341 |
| **7** | 3342 |
| **5** | 3350 |
| **10** | 3352 |
| **2** | 3357 |
| **3** | 3364 |
| **1** | 3371 |
| **1** | 3373 |
| **5** | 3401 |
| **2** | 3407 |
| **1** | 3430 |
| **2** | 3431 |
| **1** | 3434 |
| **2** | 3437 |
| **7** | 3441 |
| **4** | 3442 |
| **1** | 3444 |
| **1** | 3451 |
| **3** | 3458 |
| **13** | 3461 |
| **5** | 3463 |
| **3** | 3465 |
| **2** | 3477 |
| **35** | 3516 |
| **1** | 3517 |
| **1** | 3616 |
| **3** | 3635 |
| **12** | 3644 |
| **2** | 3658 |
| **1** | 3659 |
| **10** | 3660 |
| **12** | 3666 |
| **11** | 3669 |
| **2** | 3672 |
| **4** | 3673 |
| **5** | 3678 |
| **6** | 3683 |
| **1** | 3688 |
| **1** | 3712 |
| **1** | 3713 |
| **4** | 3714 |
| **1** | 3715 |
| **3** | 3717 |
| **5** | 3719 |
| **2** | 3730 |
| **9** | 3747 |
| **1** | 3756 |
| **2** | 3761 |
| **1** | 3777 |
| **1** | 3782 |
| **4** | 3783 |
| **1** | 3788 |
| **1** | 3797 |
| **1** | 3810 |
| **1** | 3813 |
| **1** | 3823 |
| **3** | 3824 |
| **20** | 3825 |
| **1** | 3833 |
| **10** | 3840 |
| **1** | 3842 |
| **2** | 3844 |
| **1** | 3845 |
| **6** | 3847 |
| **14** | 3851 |
| **1** | 3862 |
| **1** | 3869 |
| **2** | 3870 |
| **4** | 3871 |
| **2** | 3873 |
| **7** | 3875 |
| **10** | 3878 |
| **15** | 3880 |
| **6** | 3885 |
| **1** | 3887 |
| **1** | 3888 |
| **3** | 3891 |
| **6** | 3892 |
| **1** | 3909 |
| **1** | 3916 |
| **35** | 3921 |
| **4** | 3922 |
| **5** | 3923 |
| **7** | 3926 |
| **6** | 3927 |
| **3** | 3928 |
| **1** | 3929 |
| **1** | 3930 |
| **1** | 3934 |
| **8** | 3937 |
| **1** | 3939 |
| **1** | 3946 |
| **1** | 3951 |
| **3** | 3953 |
| **15** | 3956 |
| **9** | 3959 |
| **18** | 3960 |
| **6** | 3971 |
| **23** | 3977 |
| **1** | 3987 |
| **1** | 3992 |
| **1** | 3996 |
