# Supplementary material for: Health surveillance representative of koala (Phascolarctos cinereus) distribution in Victoria, Australia
Source: Aust Vet J. 2022 Oct 19;100(12):605–12. doi: 10.1111/avj.13208 (PMC10092863; doi:10.1111/avj.13208)
Supplement: Supplementary file 2 — Figure S2. Koala observations WHV:S submissions by postcode. [file AVJ-100-605-s003.docx]

Figure 2 Supplementary Information: Koala Observations WHV:S submissions by postcode

|  |  |
| --- | --- |
| **number of submissions** | **postcode** |
| **1** | 3995 |
| **1** | 3971 |
| **3** | 3965 |
| **9** | 3959 |
| **1** | 3956 |
| **8** | 3953 |
| **3** | 3950 |
| **3** | 3929 |
| **9** | 3927 |
| **2** | 3926 |
| **13** | 3921 |
| **1** | 3916 |
| **2** | 3915 |
| **1** | 3892 |
| **31** | 3880 |
| **3** | 3875 |
| **1** | 3871 |
| **5** | 3870 |
| **2** | 3869 |
| **2** | 3851 |
| **1** | 3850 |
| **3** | 3844 |
| **3** | 3842 |
| **1** | 3841 |
| **6** | 3840 |
| **1** | 3831 |
| **5** | 3825 |
| **1** | 3824 |
| **1** | 3823 |
| **2** | 3815 |
| **1** | 3810 |
| **1** | 3747 |
| **1** | 3717 |
| **1** | 3660 |
| **9** | 3644 |
| **14** | 3641 |
| **2** | 3442 |
| **1** | 3437 |
| **1** | 3381 |
| **1** | 3352 |
| **2** | 3350 |
| **2** | 3342 |
| **2** | 3333 |
| **1** | 3311 |
| **9** | 3305 |
| **4** | 3304 |
| **1** | 3301 |
| **1** | 3269 |
| **7** | 3234 |
| **42** | 3233 |
| **2** | 3232 |
| **1** | 3221 |
| **1** | 3213 |
| **2** | 3113 |
| **1** | 3111 |
| **1** | 2714 |
