# Supplementary material for: Health surveillance representative of koala (Phascolarctos cinereus) distribution in Victoria, Australia
Source: Aust Vet J. 2022 Oct 19;100(12):605–12. doi: 10.1111/avj.13208 (PMC10092863; doi:10.1111/avj.13208)
Supplement: Supplementary file 3 — Figure S3. SSR for each postcode. [file AVJ-100-605-s001.docx]

| Figure 3 Supplementary Information: SSR for each postcode | |
| --- | --- |
| **post code** | **SSR** |
| **3024** | 0 |
| **3097** | 0 |
| **3101** | 0 |
| **3111** | 0 |
| **3113** | 0 |
| **3114** | 0 |
| **3132** | 0 |
| **3139** | 0 |
| **3156** | 0 |
| **3158** | 0 |
| **3166** | 0 |
| **3183** | 0 |
| **3196** | 0 |
| **3199** | 0 |
| **3211** | 0 |
| **3212** | 0 |
| **3230** | 0 |
| **3231** | 0 |
| **3234** | 0 |
| **3235** | 0 |
| **3236** | 0 |
| **3238** | 0 |
| **3239** | 0 |
| **3241** | 0 |
| **3249** | 0 |
| **3260** | 0 |
| **3265** | 0 |
| **3269** | 0 |
| **3275** | 0 |
| **3277** | 0 |
| **3278** | 0 |
| **3279** | 0 |
| **3280** | 0 |
| **3281** | 0 |
| **3282** | 0 |
| **3283** | 0 |
| **3284** | 0 |
| **3285** | 0 |
| **3286** | 0 |
| **3289** | 0 |
| **3292** | 0 |
| **3294** | 0 |
| **3300** | 0 |
| **3301** | 0 |
| **3303** | 0 |
| **3311** | 0 |
| **3312** | 0 |
| **3314** | 0 |
| **3321** | 0 |
| **3331** | 0 |
| **3333** | 0 |
| **3340** | 0 |
| **3341** | 0 |
| **3357** | 0 |
| **3364** | 0 |
| **3371** | 0 |
| **3373** | 0 |
| **3381** | 0 |
| **3401** | 0 |
| **3407** | 0 |
| **3430** | 0 |
| **3431** | 0 |
| **3434** | 0 |
| **3441** | 0 |
| **3444** | 0 |
| **3451** | 0 |
| **3458** | 0 |
| **3461** | 0 |
| **3463** | 0 |
| **3465** | 0 |
| **3477** | 0 |
| **3516** | 0 |
| **3517** | 0 |
| **3616** | 0 |
| **3635** | 0 |
| **3641** | 0 |
| **3658** | 0 |
| **3659** | 0 |
| **3666** | 0 |
| **3669** | 0 |
| **3672** | 0 |
| **3673** | 0 |
| **3678** | 0 |
| **3683** | 0 |
| **3688** | 0 |
| **3712** | 0 |
| **3713** | 0 |
| **3714** | 0 |
| **3715** | 0 |
| **3719** | 0 |
| **3730** | 0 |
| **3756** | 0 |
| **3761** | 0 |
| **3777** | 0 |
| **3782** | 0 |
| **3783** | 0 |
| **3788** | 0 |
| **3797** | 0 |
| **3813** | 0 |
| **3815** | 0 |
| **3831** | 0 |
| **3833** | 0 |
| **3841** | 0 |
| **3845** | 0 |
| **3847** | 0 |
| **3850** | 0 |
| **3862** | 0 |
| **3873** | 0 |
| **3878** | 0 |
| **3885** | 0 |
| **3887** | 0 |
| **3888** | 0 |
| **3891** | 0 |
| **3909** | 0 |
| **3915** | 0 |
| **3922** | 0 |
| **3923** | 0 |
| **3928** | 0 |
| **3930** | 0 |
| **3934** | 0 |
| **3937** | 0 |
| **3939** | 0 |
| **3946** | 0 |
| **3950** | 0 |
| **3951** | 0 |
| **3960** | 0 |
| **3965** | 0 |
| **3977** | 0 |
| **3987** | 0 |
| **3992** | 0 |
| **3995** | 0 |
| **3996** | 0 |
| **3221** | 1 |
| **3233** | 1 |
| **3304** | 1 |
| **3305** | 1 |
| **3352** | 1 |
| **3660** | 1 |
| **3747** | 1 |
| **3825** | 1 |
| **3851** | 1 |
| **3921** | 1 |
| **3953** | 1 |
| **3956** | 1 |
| **2714** | 2 |
| **3213** | 2 |
| **3232** | 2 |
| **3342** | 2 |
| **3350** | 2 |
| **3437** | 2 |
| **3442** | 2 |
| **3644** | 2 |
| **3717** | 2 |
| **3810** | 2 |
| **3823** | 2 |
| **3824** | 2 |
| **3840** | 2 |
| **3842** | 2 |
| **3844** | 2 |
| **3869** | 2 |
| **3870** | 2 |
| **3871** | 2 |
| **3875** | 2 |
| **3880** | 2 |
| **3892** | 2 |
| **3916** | 2 |
| **3926** | 2 |
| **3927** | 2 |
| **3929** | 2 |
| **3959** | 2 |
| **3971** | 2 |
